# AKR1C3 and Its Transcription Factor HOXB4 Are Promising Diagnostic Biomarkers for Acute Myocardial Infarction

**DOI:** 10.3389/fcvm.2021.694238

**Published:** 2021-09-09

**Authors:** Jingjing Liang, Yue Cao, Mingli He, Weiwen Li, Guolin Huang, Tianyi Ma, Meijun Li, Yuli Huang, Xiaohui Huang, Yunzhao Hu

**Affiliations:** ^1^Department of Cardiology, Shunde Hospital, Southern Medical University, Foshan, China; ^2^The Second School of Clinical Medicine, Southern Medical University, Guangzhou, China; ^3^Department of Cardiology, Affiliated Haikou Hospital of Xiangya Medical College, Central South University, Haikou, China

**Keywords:** AKR1C3, HOXB4, ferroptosis, acute myocardial infarction, diagnosis

## Abstract

**Background:** A recent study disclosed that ferroptosis was an important myocyte death style in myocardial infarction (MI). However, the diagnostic value of ferroptosis regulators and correlated underlying mechanisms in acute myocardial infarction (AMI) remain unknown.

**Methods:** Bioinformatical analyses were conducted to identify the candidate biomarkers for AMI, and the collected local samples were used to validate the findings *via* real-time quantitative PCR. Bioinformatical analysis and luciferase reporter assay were implemented to identify the transcriptional factor. Transient transfection and ferroptosis characteristic measurement, including glutathione peroxidase 4, malondialdehyde, iron, and glutathione, was performed to verify the ability of the candidate gene to regulate the ferroptosis of cardiomyocytes. A meta-analysis was conducted in multiple independent cohorts to clarify the diagnostic value.

**Results:** A total of 121 ferroptosis regulators were extracted from previous studies, and aldo-keto reductase family 1 member C3 (AKR1C3) was significantly downregulated in the peripheral blood samples of AMI cases from the analysis of GSE48060 and GSE97320. HOXB4 served as a transcriptional activator for AKR1C3 and could suppress the ferroptosis of the H9C2 cells treated with erastin. Besides this, peripheral blood samples from 16 AMI patients and 16 patients without coronary atherosclerotic disease were collected, where AKR1C3 and HOXB4 both showed a high diagnostic ability. Furthermore, a nomogram including HOXB4 and AKR1C3 was established and successfully validated in six independent datasets. A clinical correlation analysis displayed that AKR1C3 and HOXB4 were correlated with smoking, CK, CK-MB, and N-terminal-pro-B-type natriuretic peptide.

**Conclusion:** Taken together, this study demonstrates that AKR1C3 and HOXB4 are promising diagnostic biomarkers, providing novel insights into the ferroptosis mechanisms of AMI.

## Introduction

Cardiovascular disease has become a major contributor to the global burden of diseases with significant morbidity and mortality worldwide (1). Acute myocardial infarction (AMI), commonly defined as myocardial necrosis due to an imbalance between oxygen supply and demand, is the leading cause of death in cardiovascular disease (2). AMI is caused by the rupture of the instable atherosclerotic plaques, which occur with a sudden onset and may cause prehospital cardiac arrest, thus making rapid assessment, early diagnosis, and initial treatment beneficial to improve the prognosis of patients (3). Guidelines from the European Society of Cardiology (ESC) recommended that assessment and treatment, including thrombolysis and surgery, should be administered within 2–24 h. In the clinic, ~25% of patients with AMI do not show typical symptoms and signs, and 50% electrocardiogram (ECG) lacked the representative manifestation of AMI (2). Therefore, sensitive markers of cardiac ischemic damage could help identify clinically meaningful points of timely diagnosis and prevention of myocardial infarction.

The diagnosis and prognosis of heart disease are related to the degree of myocardial injury. After half a century of research on different types and detection methods of myocardial injury markers, the diagnosis of AMI is based on ECG and serum markers of myocardial injury that include cTnI/T and CKMB, playing an important role in reducing the mortality and prognosis by myocardial damage. However, in practice, the sensitivity and specificity of these diagnostic criteria are limited and may lead to a misdiagnosis (4). In recent years, new diagnostic markers of myocardial infarction have attracted more and more attention. The current study suggested new biomarkers of diagnosis by recognizing gene expression profiles, providing a potential therapeutic target with differentially expressed genes (5).

Ferroptosis is a type of programmed cell death that is distinct from apoptosis and autophagy and is driven by the excessive accumulation of iron-dependent lipid reactive oxygen species (ROS) in the cells (6). In a recent study, several factors that regulate ferroptosis have been identified in cardiovascular disease—for example, the transcription factor erythroid 2-related factor 2 is a key regulator of oxidative stress response, which plays a key role in ferroptosis, becoming a significant target in the therapy of tumor and cardiovascular and cerebrovascular diseases (7). Other genes like acyl-CoA synthetase long-chain family member 4 and glutathione peroxidase 4 (GPX4) have been proven to be the contributor of lipid peroxidation and participated in the iron death of cardiomyocytes (8). Ferroptosis is an important cause of cell death in myocardial infarction area, and Baba *et al*. have demonstrated that mTOR can inhibit the iron death process of myocardial cells by regulating ROS and iron metabolism in adult mice (9). However, the potential genes regulating ferroptosis in identifying AMI remain indeterminate.

In this study, we analyzed a microarray of AMI compared with the normal on the basis of known ferroptosis regulators *in silico* (6). Validation in both external data set and human blood samples was conducted in our study. Besides this, to explore underlying molecular mechanisms, we implemented bioinformatical analysis and luciferase reporter array to predict and validate the transcription factor. The ferroptosis association of the novel transcriptional factor was also detected through multiple ferroptosis characteristic measurement. To improve the diagnostic accuracy, a nomogram was established and validated, helping clinicians understand the risk mode more easily. The diagnostic ability of the established nomogram was tested in six independent datasets *via* meta-analysis. These may do help to determine novel diagnostic biomarkers in AMI, also disclosing latent mechanisms and providing potential therapeutic targets.

## Materials and Methods

### Ethics

The studies involving human participants were reviewed and approved by the Ethics Committee of Shunde Hospital of Southern Medical University. All participants signed an informed consent to take part in the study. The ethics review material is provided in Supplementary Material 1.

### Data Collection and Processing

The microarray datasets GSE48060 and GSE97320 were all downloaded from Gene Expression Omnibus (GEO, https://www.ncbi.nlm.nih.gov/geo/). Affy and sva packages of R were implemented for robust multi-array average (RMA) and surrogate variable analysis (SVA) to remove the batch effect as possible. Limma package was used to detect the differentially expressed genes (DEGs), and |log fold change, FC| >0.7 and adjusted *p*-value < 0.05 were regarded to be statistically significant. Other GEO datasets, like GSE34198, GSE42148, GSE60993, and GSE61144, were directly downloaded as gene expression matrix from the GEO website.

### Sample Collection

The peripheral blood samples from 16 AMI and 16 control samples were collected from the Department of Cardiology in Shunde Hospital of Southern Medical University from November 2020 to January 2021. The peripheral blood samples were all collected within 24 h after AMI onset, and informed consents were signed. The diagnostic criteria of AMI were based on the latest clinical guidelines (10). The control samples were also collected after excluding the cases with a previous history of cardiovascular diseases, malignant tumor, severe infection, hepatorenal insufficiency, and other factors which might affect the test results. The medical history and the results of the first blood test after admission were also collected.

### Validation of the Diagnostic Value

Differential methods were used to measure the diagnostic value. Receiver operating characteristic (ROC) analyses were conducted *via* the pROC R package. The Rms R package was used to draw the calibration and decision curve analysis (DCA) curves as well as nomogram.

### Meta-Analysis

A meta-analysis was conducted to pool the odds ratios (ORs) of each study with meta package (11) of R. When the heterogeneity test indicated statistical significance (*p* < 0.1), random effects model would be applied; otherwise, a fixed effect model would be adopted.

### Co-expression Network Construction

The weighted gene co-expression network was constructed by the WGCNA package of R. The minimal gene size was set as 30 to construct a dendrogram. Module eigengenes were identified as a cluster of genes with high correlation. The interesting gene module was picked out when the module had the highest correlation with a logical score.

### GO Enrichment Analysis

Gene Ontology (GO) analysis was conducted with clusterProfile R package after transforming the gene symbol into Entrez id *via* the org.Hs.eg.db package.

### PPI Network Construction

The protein–protein interaction (PPI) information was obtained from STRING database (https://string-db.org/), and the interaction score was set as 0.4. Cytoscape software (version 3.8.0) was used for network visualization.

### Gene Set Enrichment Analysis

Gene Set Enrichment Analysis (GSEA) software (version 4.1.0) was downloaded from the GSEA official website (https://www.gsea-msigdb.org/gsea/index.jsp). C2 cp kegg v7.2 was obtained from Molecular Signatures Database (https://www.gsea-msigdb.org/gsea/msigdb/index.jsp) and chosen as the reference signature. Single-gene GSEA strategy meant that we divided all samples into two groups according to the median expression value of the gene and then performed GSEA.

### Potential Compounds Targeting HOXB4–AKR1C3 Axis Prediction

CMap database was used for the prediction of potential compounds with *p* < 0.001. Bubble charts were drawn to visualize the result by means of ggplot2 package.

### Cell Culture and Treatment

The 293T and H9C2 cell lines were obtained from Cell Bank, Chinese Academy of Sciences in Shanghai, China. The cells were cultured in Dulbecco's modified Eagle's medium, which was supplemented with 10% fetal bovine serum and 1% antibiotics (Gibco, China). The cells were cultured in a humidified atmosphere with 5% CO_2_ at 37°C. After treating with 5 nM erastin (MedChemExpress, USA) for 24 h (8), the H9C2 cells were collected.

### Total RNA Isolation and RT-qPCR

We collected 6 ml of peripheral blood from each subject and stored the sample in EDTA anticoagulant tubes. The total RNA of the blood samples was extracted with MolPure Blood RNA Kit (YASHEN, China). The total RNA of the cells was isolated by MolPure TRIeasy Plus Total RNA Kit (YASHEN, China). The cDNA was synthesized through Hifair III One Step RT-qPCR Probe Kit (YASHEN, China). RT-qPCR was performed with Hieff qPCR SYBR Green Master Mix (YASHEN, China). The primers were designed as shown in Supplementary Table 11. The expression value was measured using 2^−ΔΔCT^ method, where CT meant threshold cycle.

### Luciferase Reporter Assay

293T cells were co-transfected with an aldo-keto reductase family 1 member C3 (AKR1C3) promoter–luciferase reporter vector and a HOXB4 expression vector (0.8 μg, HANBIO, China) with the company of LipoFiterTM Liposomal Transfection Reagent (2 μl, HANBIO, China). We changed the fresh medium every 6 h, and after 24 h, the cells were collected for testing. The luciferase activity was evaluated with Dual-Luciferase Reporter Assay System (Promega). The fluorescence intensity was measured by Synergy HTX (BioTek, USA).

### HOXB4 Overexpression Through Transient Transfection

The H9C2 cells with 50–60% confluence under no-serum medium in a six-well plate were used for transfection. Subsequently, the plasmids of pCMV5-HOXB4 (5 μg) were transfected into H9C2 cell lines for 24 h according to the instructions of the manufacturer of PureFection™ Nanotechnology-based Transfection Reagent (System Biosciences, USA). Western blot (WB) was used to detect whether the HOXB4-overexpression cells were successfully established.

### Ferroptosis Marker Measurement

The relative levels of malondialdehyde (MDA), iron, and glutathione (GSH) were detected *via* Lipid Peroxidation Assay Kit (Abcam, USA), Iron Assay Kit (Abcam, USA), and GSH Colorimetric Detection Kit (Thermo Fisher, USA), respectively. We followed the protocols from the manufacturer as to how to conduct the series of experiments.

### Western Blot

Total protein was obtained from the lysed cells with RIPA buffer (Thermo Scientific, USA), and the protein concentration was quantified with BCA Protein Assay Kit (Abcam, USA). Protein electrophoresis and transferring to nitrocellulose membrane followed the routine protocols described in our previous research. The membrane was blocked with bovine serum albumin (5%) for 1 h, and the membrane was hatched overnight with the primary antibodies against HOXB4, AKR1C3, GPX4, and β-actin (Abcam, USA). The membrane was washed with TBST for five times and incubated with the second antibodies at 37°C for 1 h. Enhanced Chemiluminescence Reaction Solution (Pierce, Rockford, IL, USA) was added to the membrane for 1 min. We chose β-actin as the internal reference. The protein images were explicated with ImageJ2x, and Gelpro 32 software was used to analyze the gray values.

### Statistical Power Analyses

In order to detect whether the sample size of the training dataset (31 AMI vs. 21 control) was enough to achieve a statistically significant conclusion, the statistical power was calculated based on the known ROC by utilizing PASS 15 software. As a general rule, the sample size was considered as within acceptance when the power is >0.9.

## Results

### AKR1C3 Was a Ferroptosis-Related Biomarker for AMI Diagnosis

**Figure 1:**
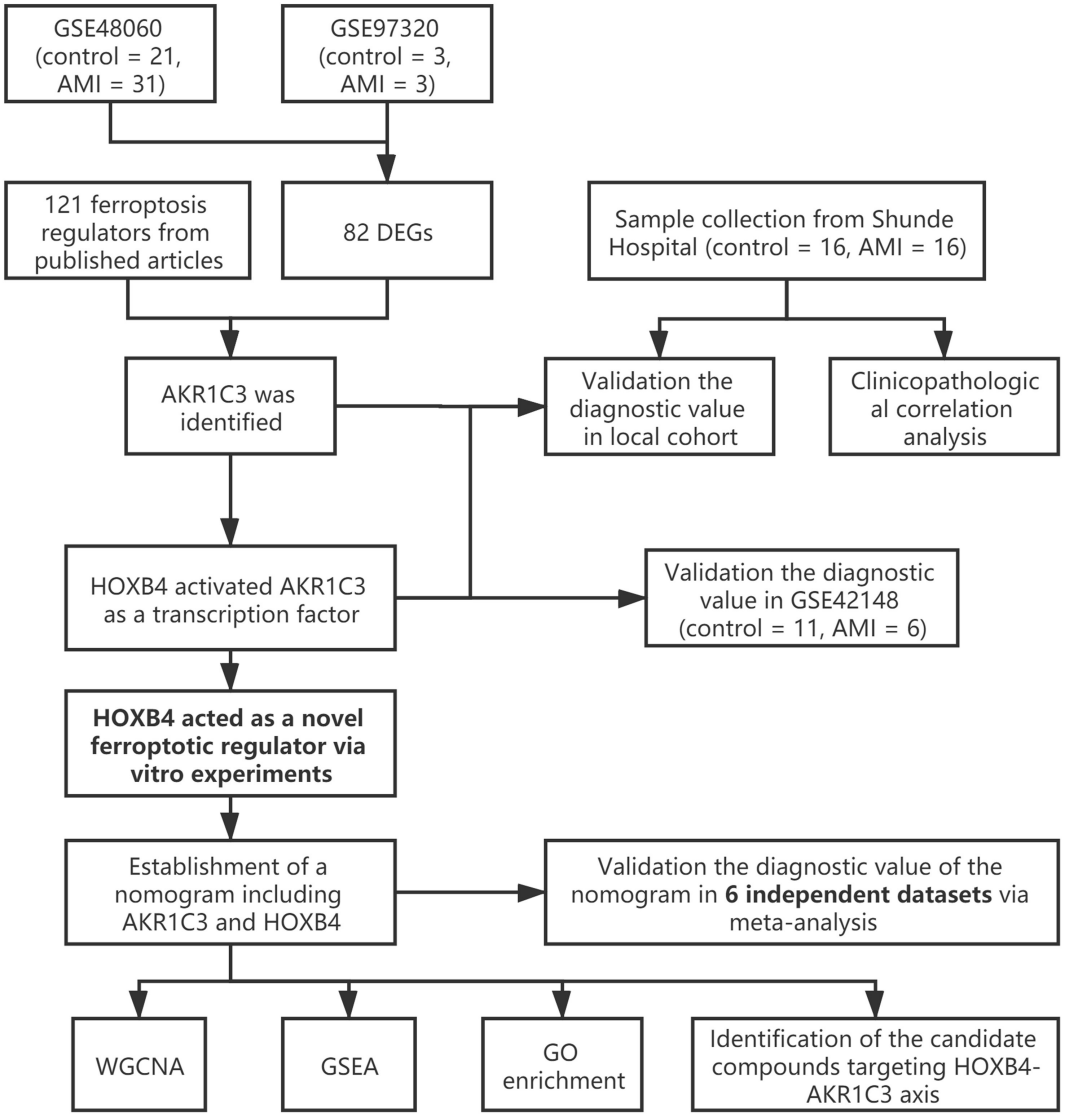
The workflow of the present study.

**Figure 2:**
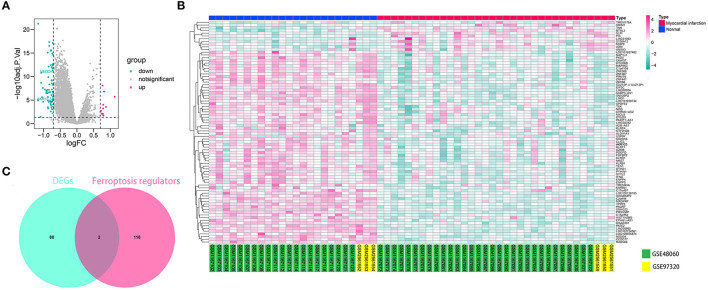
AKR1C3 was differentially expressed in acute myocardial infarction (AMI) samples. **(A)** Volcano map indicating the 82 differentially expressed genes (DEGs) between normal and AMI samples. **(B)** Heat map showing the 82 DEGs. **(C)** Among 120 ferroptosis-related genes, AKR1C3 and NEDD4 were differentially expressed.

**Figure 3:**
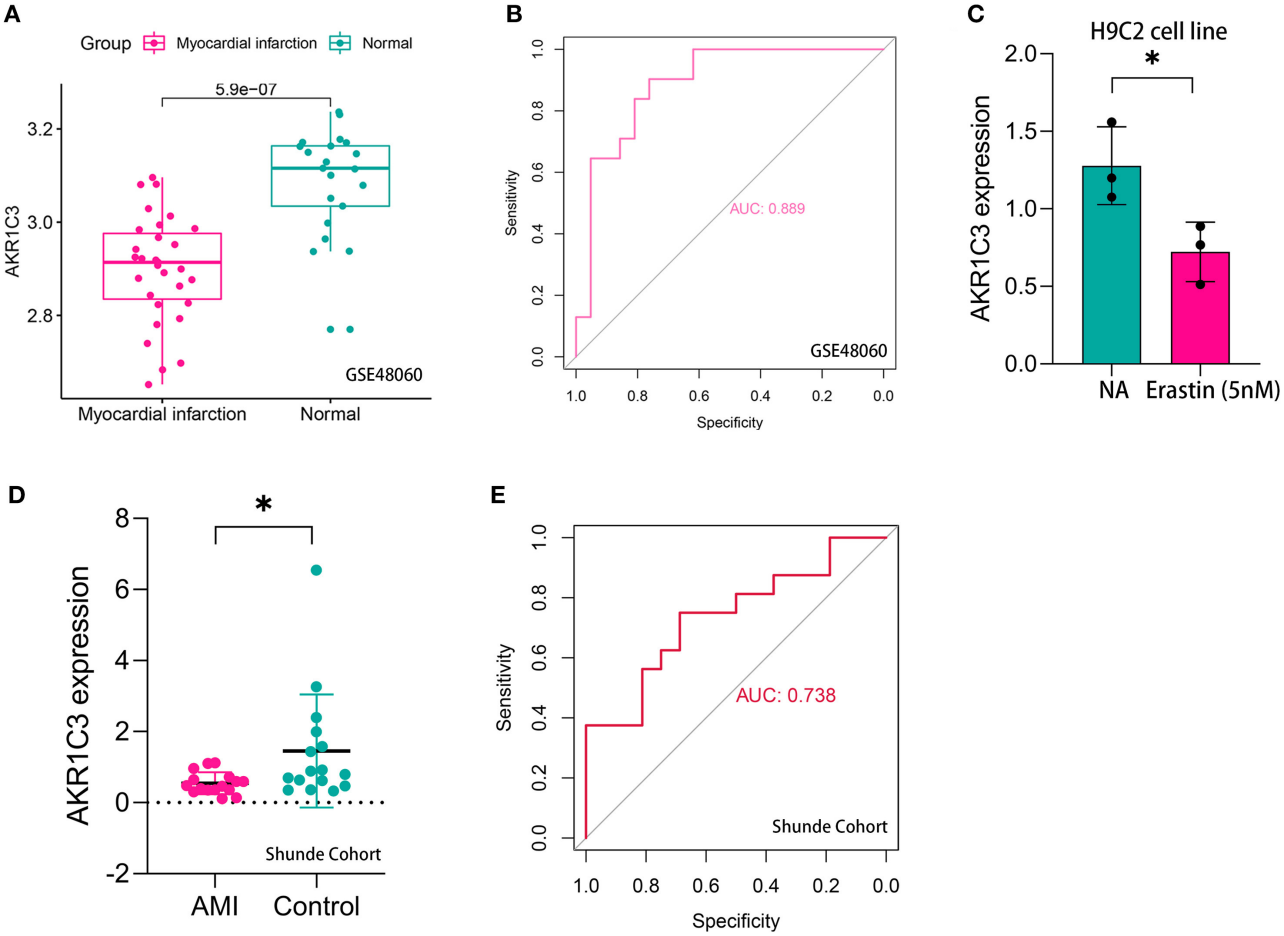
AKR1C3 was a ferroptosis-related biomarker for acute myocardial infarction (AMI) diagnosis. **(A)** AKR1C3 was significantly downregulated in AMI samples of the GSE48060 cohort. **(B)** The receiver operating characteristic (ROC) curve for the diagnostic value of AKR1C3 in GSE48060. **(C)** AKR1C3 was downregulated in H9C2 cells treated with 5 nM erastin. **(D)** AKR1C3 was significantly downregulated in AMI samples of the local cohort. **(E)** The ROC curve for the diagnostic value of AKR1C3 in the local cohort. *p < 0.05.

The workflow of the present study is shown in Figure 1. First, the raw CEL files of GSE48060 and GSE97320 were downloaded from GEO and normalized through RMA and SVA. GSE48060 contained 21 peripheral blood samples of healthy donors and 31 peripheral blood samples of AMI patients, and GSE97320 included three control samples and three AMI samples. Then, limma R package was used for genomic divergence detection, and 82 DEGs were screened with |logFC| >0.7 and adjusted *p*-value < 0.05 filtering (Supplementary Table 1; Figure 2A). The expression level of 82 DEGs in each sample was visualized *via* heat map (Figure 2B). A total of 120 known ferroptosis regulators were collected from previous studies (6), and AKR1C3 and NEDD4 were found to be differentially expressed (Figure 2C). A recent study has reported that AKR1C3 might serve as a diagnosis marker for AMI *via* a machine learning algorithm (12). However, the conclusion was based on a single dataset, and further exploration of the underlying mechanisms was lacking. Therefore, we collected six datasets, which all included the peripheral blood samples from control cases and AMI patients (Table 1). The local samples, including 16 control and 16 AMI samples, were also collected from Shunde Hospital of Southern Medical University. H9C2 cells, which were cardiac myocytes of mice, were cultured and treated with erastin to determine the correlation of ferroptosis in cardiomyocytes. Erastin, which was able to modulate VDAC2/VDAC3 and system xc-, could induce ferroptosis (13). AKR1C3 was significantly downregulated in AMI samples in the GSE48060 dataset (*p* < 0.05, Figure 3A) and Shunde cohort (*p* < 0.05, Figure 3D) with Wilcoxon signed-rank test and *t*-test, respectively. The ROC-indicated areas under the curve (AUCs) were 0.889 (Figure 3B) and 0.738 (Figure 3E) in GSE48060 and Shunde cohort, showing that AKR1C3 has a high efficacy to distinguish AMI from control cases. The expression of AKR1C3 was inhibited in H9C2 cells after treating with erastin (*p* < 0.05, Figure 3C), indicating that AKR1C3 might be involved in the process of ferroptosis in cardiac myocytes.

**Table 1:** The microarray datasets enrolled in the study.

**Datasets**	**Platform**	**Sample size (control/AMI)**	**Region**
GSE48060	(HG-U133_Plus_2) Affymetrix Human Genome U133 Plus 2.0 Array	52 (21/31)	America
GSE97320	(HG-U133_Plus_2) Affymetrix Human Genome U133 Plus 2.0 Array	6 (3/3)	China
GSE42148	Agilent-028004 SurePrint G3 Human GE 8 × 60K Microarray (feature number version)	17 (11/6)	Indian
GSE34198	Illumina Human-6 v2.0 expression beadchip	97 (48/49)	Czech
GSE60993	Illumina HumanWG-6 v3.0 expression beadchip	24 (7/17)	South Korea
GSE61144	Sentrix Human-6 v2 expression beadchip	17 (10/7)	South Korea

### HOXB4 Served as a Transcription Factor to AKR1C3

**Figure 4:**
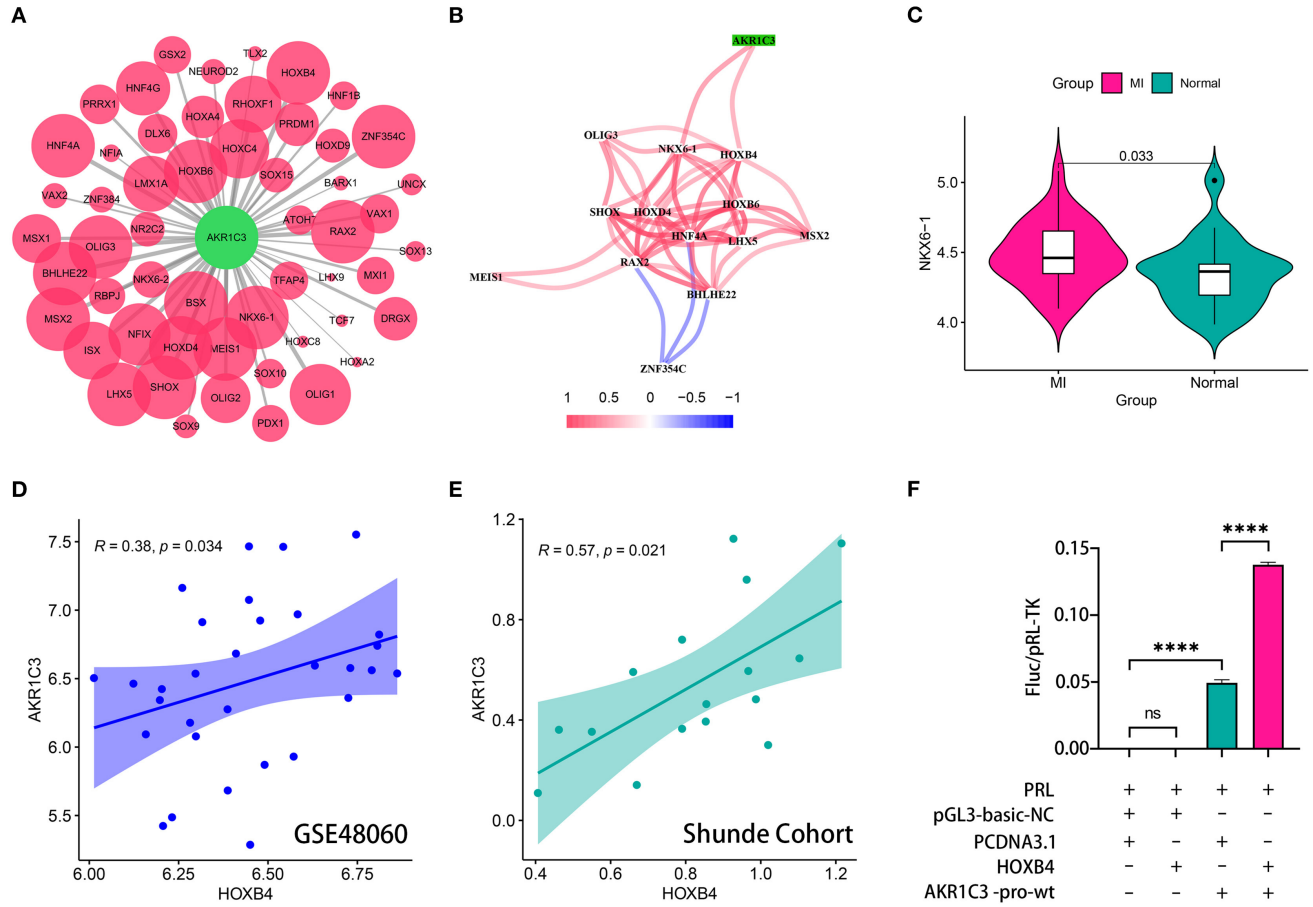
HOXB4 served as a transcription factor for AKR1C3. **(A)** Prediction of the potential transcription factor of AKR1C3. The size of the circle represents the predicted possibility. **(B)** AKR1C3 was significantly correlated with HOXB4 and NKX6-1. **(C)** NKX6-1 was upregulated in acute myocardial infarction samples. **(D,E)** The expression values of AKR1C3 and HOXB4 were tightly associated in the GSE48060 **(D)** and Shunde **(E)** cohorts. **(F)** The results of the luciferase reporter assay. ****p < 0.0001; ns, not significant.

The sequence from 2,000 bp upstream to 500 bp downstream of the transcription start site of AKR1C3 was obtained from UCSC Genome Browser (http://genome.ucsc.edu/). We utilized TFBSTools, JASPAR2020, and Biostrings R packages to predict the possible transcription factors (Supplementary Table 2; Figure 4A). The Spearman correlation analysis indicated that NKX6-1 and HOXB4 were positively correlated with AKR1C3 with *r* > 0.3 and *p* < 0.05 filtering (Supplementary Table 3; Figure 4B). However, NKX6-1 was upregulated in AMI samples (*p* < 0.05, Figure 4C) though AKR1C3 was downregulated, so HOXB4 was then chosen as the potential transcription factor. HOXB4 had a significantly positive correlation with AKR1C3 both in GSE48060 (Spearman *r* = 0.38, *p* < 0.05, Figure 4D) and local cohort (Spearman *r* = 0.57, *p* < 0.05, Figure 4E). Subsequently, we validated that HOXB4 could interact with the promoter of AKR1C3 *via* luciferase reporter assay (Supplementary Table 4; Figure 4F).

### HOXB4 Was a Ferroptosis-Related Biomarker for AMI Diagnosis

**Figure 5:**
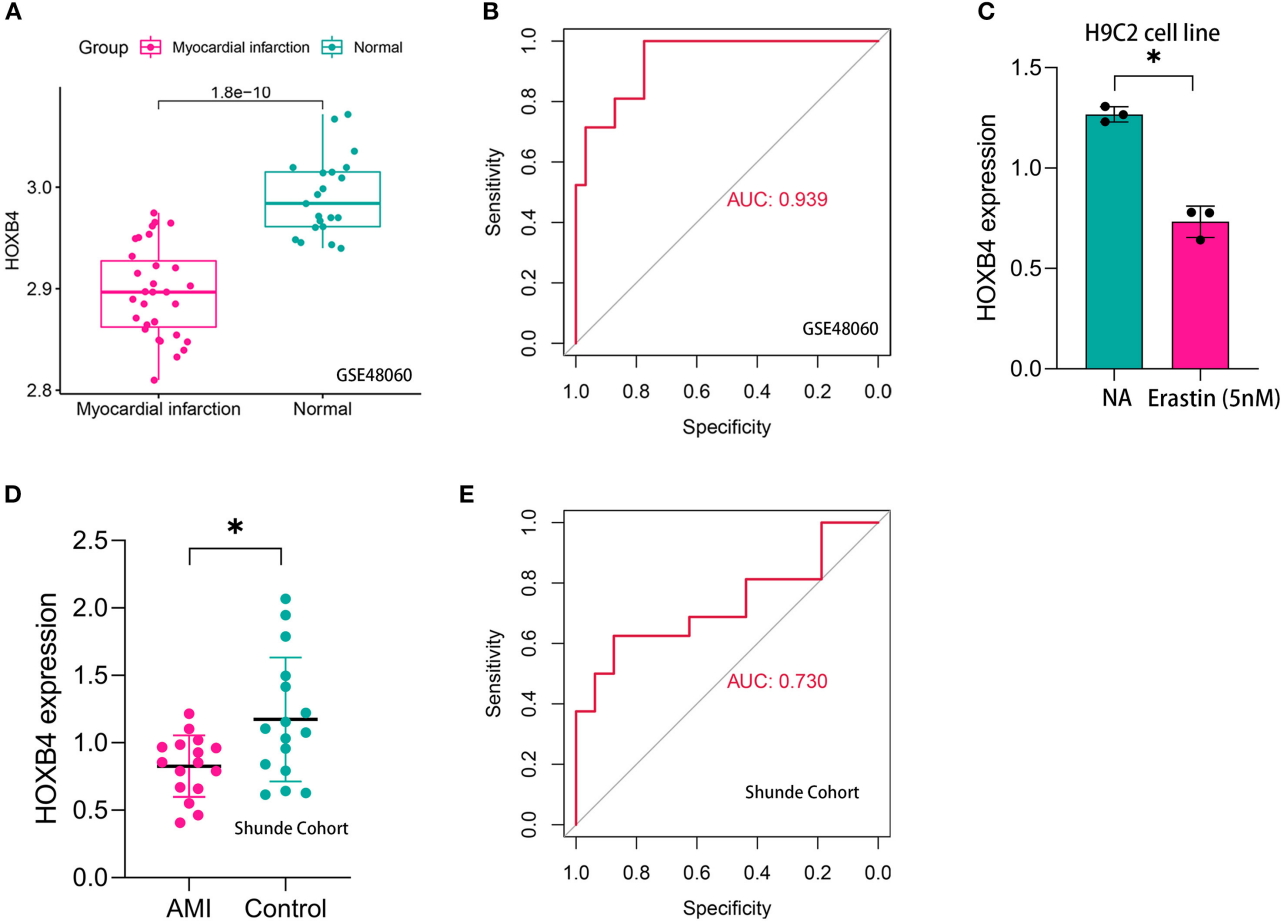
HOXB4 was a ferroptosis-related biomarker for acute myocardial infarction (AMI) diagnosis. **(A)** HOXB4 was significantly downregulated in the AMI samples of the GSE48060 cohort. **(B)** The receiver operating characteristic (ROC) curve for the diagnostic value of HOXB4 in GSE48060. **(C)** HOXB4 was downregulated in H9C2 cells treated with 5 nM erastin. **(D)** HOXB4 was significantly downregulated in the AMI samples of the local cohort. **(E)** The ROC curve for the diagnostic value of HOXB4 in the local cohort. *p < 0.05.

Compared with the samples collected from the controls, HOXB4 was significantly downregulated in the peripheral blood samples of AMI patients both in GSE48060 (*p* < 0.001, Figure 5A) and Shunde cohort (*p* < 0.05, Figure 5D) *via* Wilcoxon signed-rank test and Student's *t*-test, respectively. HOXB4 also showed a high diagnostic value in GSE48060 (AUC = 0.939, Figure 5B) and Shunde cohort (AUC = 0.730, Figure 5E) by means of ROC analysis. Besides this, we found that HOXB4 was differentially expressed in H9C2 cells treated with erastin (*p* < 0.05, Figure 5C).

### The Overexpression of HOXB4 Suppressed the Ferroptosis of Cardiomyocytes

**Figure 6:**
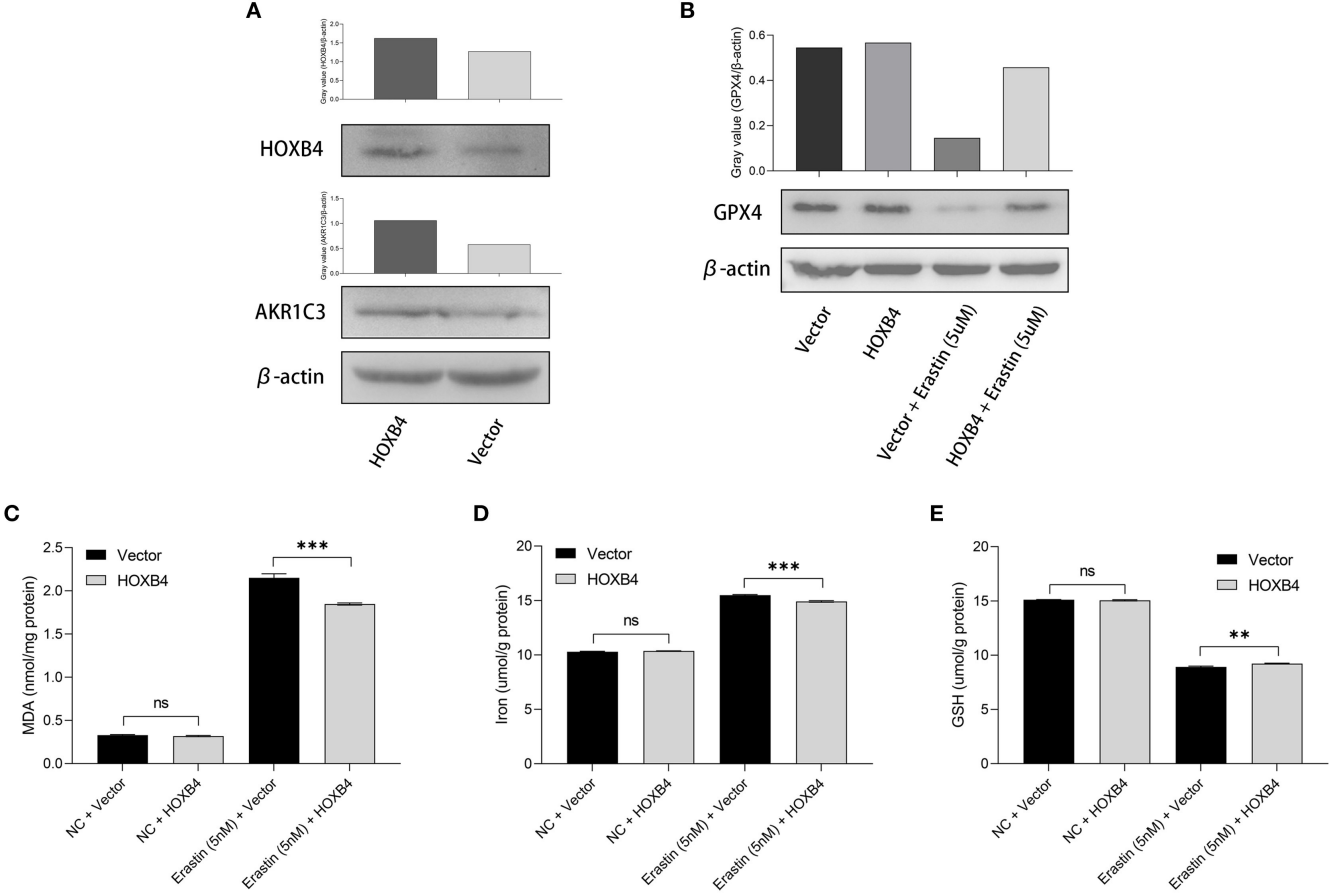
The upregulation of HOXB4 suppressed ferroptosis in H9C2 cells. **(A)** A HOXB4-overexpression H9C2 cell line was successfully constructed *via* transient transfection, and AKR1C3 was upregulated in the cells with overexpressed HOXB4. **(B)** GPX4 was significantly upregulated in the HOXB4-overexpression cells with erastin treatment. **(C–E)** The overexpression of HOXB4 was significantly associated with the level of malondialdehyde **(C)**, iron **(D)**, and glutathione **(E)**. **p < 0.01; ***p < 0.001; ns, not significant.

Utilizing the transient transfection experiments, H9C2 cell line with HOXB4 overexpression was constructed (Figure 6A). We also found that the expression of AKR1C3 was upregulated in the HOXB4-overexpression cells, re-validating the transcriptional interaction (Figure 6A). Subsequently, different ferroptosis measurements were conducted after the 5-nM-erastin treatment of the H9C2 cells, including GPX4 (Figure 6B), MDA (*p* < 0.001, Figure 6C), iron level (*p* < 0.001, Figure 6D), and GSH (*p* < 0.01, Figure 6E), by WB and enzyme-linked immunosorbent assay. The findings mentioned above suggested that HOXB4 could inhibit ferroptosis in the cardiomyocytes.

### Construction of a Diagnostic Nomogram Including AKR1C3 and HOXB4

**Figure 7:**
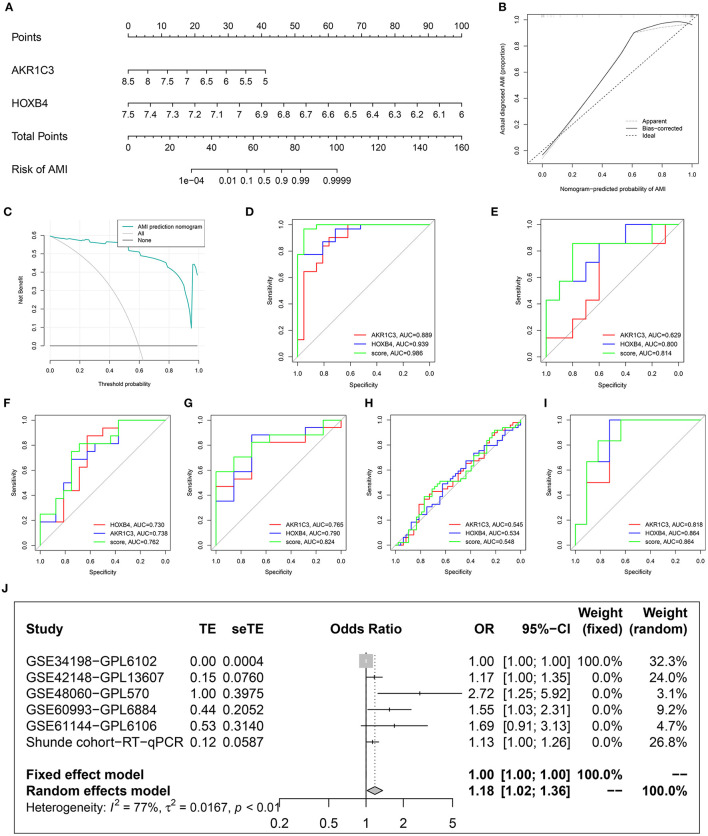
Construction and validation of a diagnostic model. **(A)** The established nomogram. **(B)** Calibration plot. **(C)** Decision curve analysis. **(D–I)** Receiver operating characteristic analysis in GSE48060 **(D)**, GSE61144 **(E)**, Shunde cohort **(F)**, GSE60993 **(G)**, GSE34198 **(H)**, and GSE42148 **(I)**. **(J)** Meta-analysis.

To improve the diagnostic efficacy, HOXB4 and AKR1C3 were both used for the establishment of a diagnostic model by multivariate logical regression. The logical regression model was constructed as follows: logical score = 140.931 - 3.099*EXP(AKR1C3) - 17.572*EXP(HOXB4), where EXP meant the mRNA expression value of the gene. To help clinicians better understand the diagnostic model, a nomogram was drawn (Figure 7A), which was based on the GSE48060 dataset. The calibration plot (Figure 7B) and DCA (Figure 7C) indicated that the nomogram could predict the occurrence of AMI with a high efficacy.

To explore whether the sample size is big enough, PASS 15 was used to calculate the statistical power of the training dataset. The analyses indicated that the power for the logical score, AKR1C3, and HOXB4 were all 1.00, showing that the sample size was reasonable.

### Validation of the Diagnostic Model in Multiple Datasets

After removing the datasets with <5 samples in the control or AMI group to ensure the statistical efficiency of logical regression, four datasets, including GSE34198, GSE42148, GSE60993, and GSE61144, were selected for external validation. The details of the GEO datasets are shown in Table 1. GSE48060 was set as the training dataset, and the collected samples from a local hospital were also used for validation. The ROC analysis indicated that the AUCs of the logical model were mostly over 0.7 except for GSE34198 (Figures 7D–I). Sequencing error and expression variance in different populations might account for the difference. Besides these, the AUCs of logical score were higher than those of HOXB4 and AKR1C3 in all datasets, implying that the combination of AKR1C3 and HOXB4 was a useful strategy. To detect whether the logical model could diagnose AMI, a meta-analysis was implemented to combine the ORs in each cohort. As shown in Figure 7J, the meta-analysis showed that the cases with a relatively high logical score were more likely to be diagnosed as AMI (pooled OR = 1.18; 95%CI, 1.02–1.36).

### Clinical Relevance

**Figure 8:**
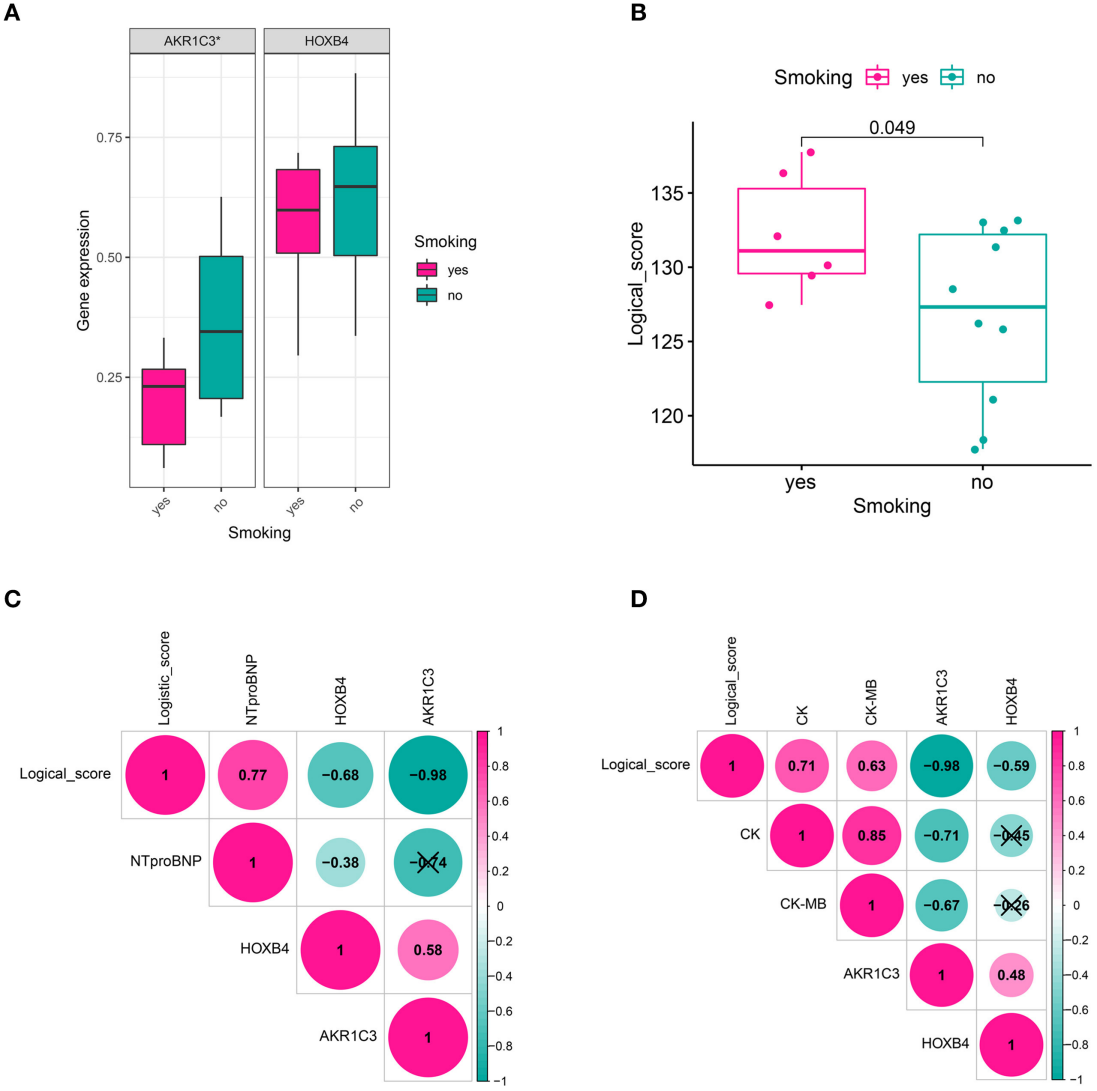
Clinical relevance. **(A)** AKR1C3 was downregulated in acute myocardial infarction (AMI) patients with smoking history. **(B)** The cases with smoking history had a higher logical score, which meant that these people were more likely to be diagnosed as AMI. **(C)** HOXB4 and the logical score were significantly correlated with the value of NTproBNP. **(D)** AKR1C3 and the logical scores had a significant association with CK and CK-MB. NTproBNP, N-terminal-pro-B-type natriuretic peptide; CK, creatine kinase; CK-MB, creatine kinase MB fraction; *p < 0.05. ns, not significant.

We also collected the clinical information of 16 patients in a local hospital. It was found that AKR1C3 was significantly correlated with smoking (Wilcoxon *p* < 0.05, Figure 8A), CK (creatine kinase, Spearman *r* = −0.71, *p* < 0.05, Figure 8D), and CK-MB (Spearman *r* = −0.67, *p* < 0.05, Figure 8D), while HOXB4 was significantly correlated with N-terminal-pro-B-type natriuretic peptide (NTproBNP, Spearman *r* = −0.38, *p* < 0.05, Figure 8C). The correlation of the logical score with smoking (Wilcoxon *p* < 0.05, Figure 8B), NTproBNP (Spearman *r* = 0.77, *p* < 0.05, Figure 8C), CK (Spearman *r* = 0.71, *p* < 0.05, Figure 8D), and CK-MB (Spearman *r* = 0.63, *p* < 0.05, Figure 8D) was also significant.

### Weighted Gene Co-expression Network Analysis

**Figure 9:**
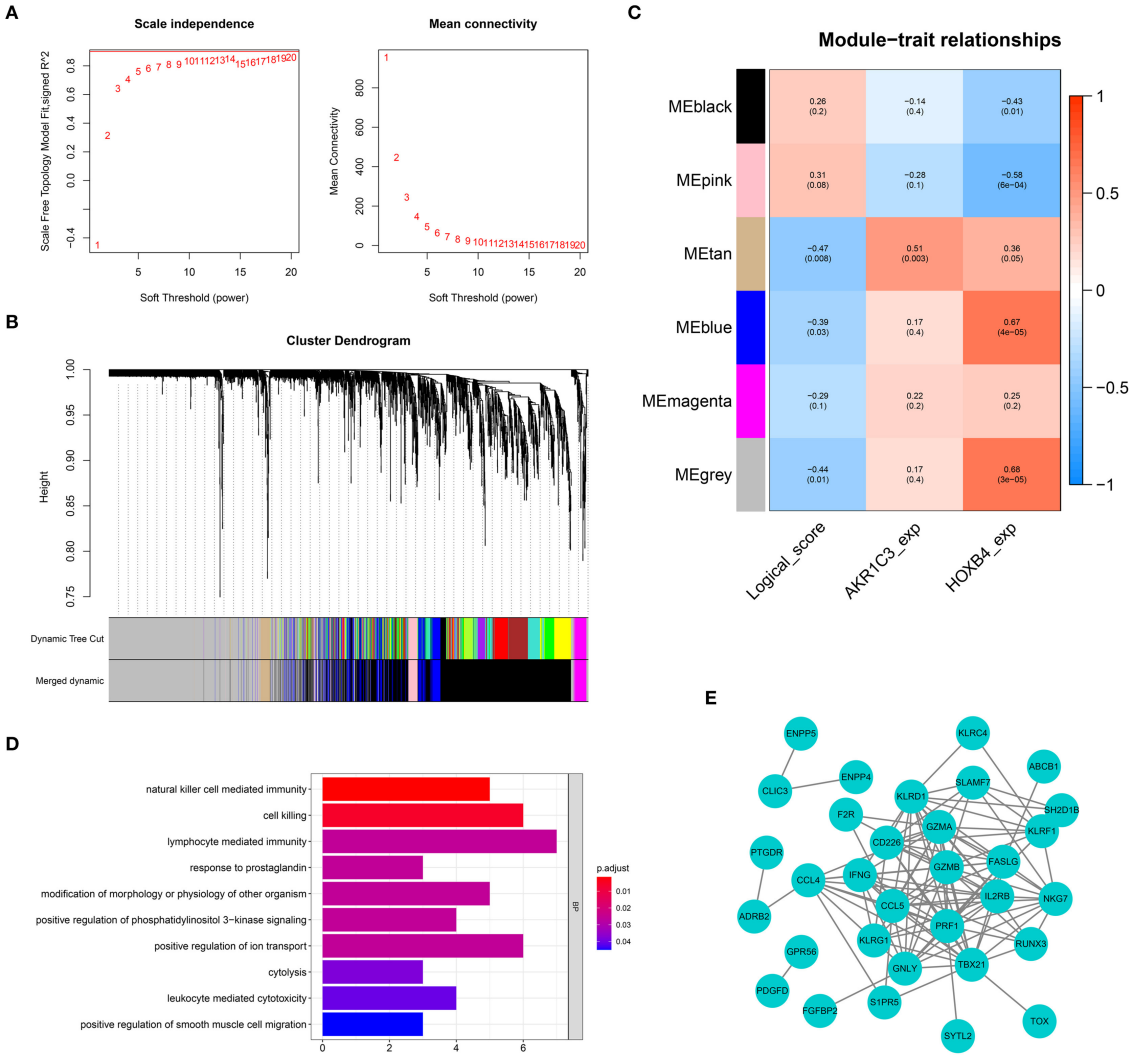
Weighted gene co-expression network analysis. **(A)** The optimal soft-thresholding value was equal to 19. **(B)** The genes were divided into six different gene modules. **(C)** The heat map showing the correlation between logical score and each module. **(D)** Gene Ontology enrichment. **(E)** Protein–protein interaction network construction.

To screen the genes related with logical score, a co-expression network was established based on the expression matrix of AMI samples from GSE48060. A total of 2,858 DEGs were extracted with |logFC| >0.3 and adjusted *p*-value < 0.05 filtering for co-expression network construction (Supplementary Table 5). The optimal soft-thresholding value was set as 19 (Figure 9A). Six different gene modules were identified (Figure 9B), and tan module had the highest correlation with logical score (*r* = −0.47, *p* < 0.01, Figure 9C). The correlation between each gene and gene module is shown in Supplementary Table 6. A total of 63 genes in tan module were considered to be closely correlated with logical score. GO analysis showed that the genes in tan module were mostly enriched in immune-related functions, like natural killer cell-mediated immunity and lymphocyte-mediated immunity (Figure 9D). To explore the interaction of these 63 genes, a PPI network was established (Figure 9E).

### GSEA

**Figure 10:**
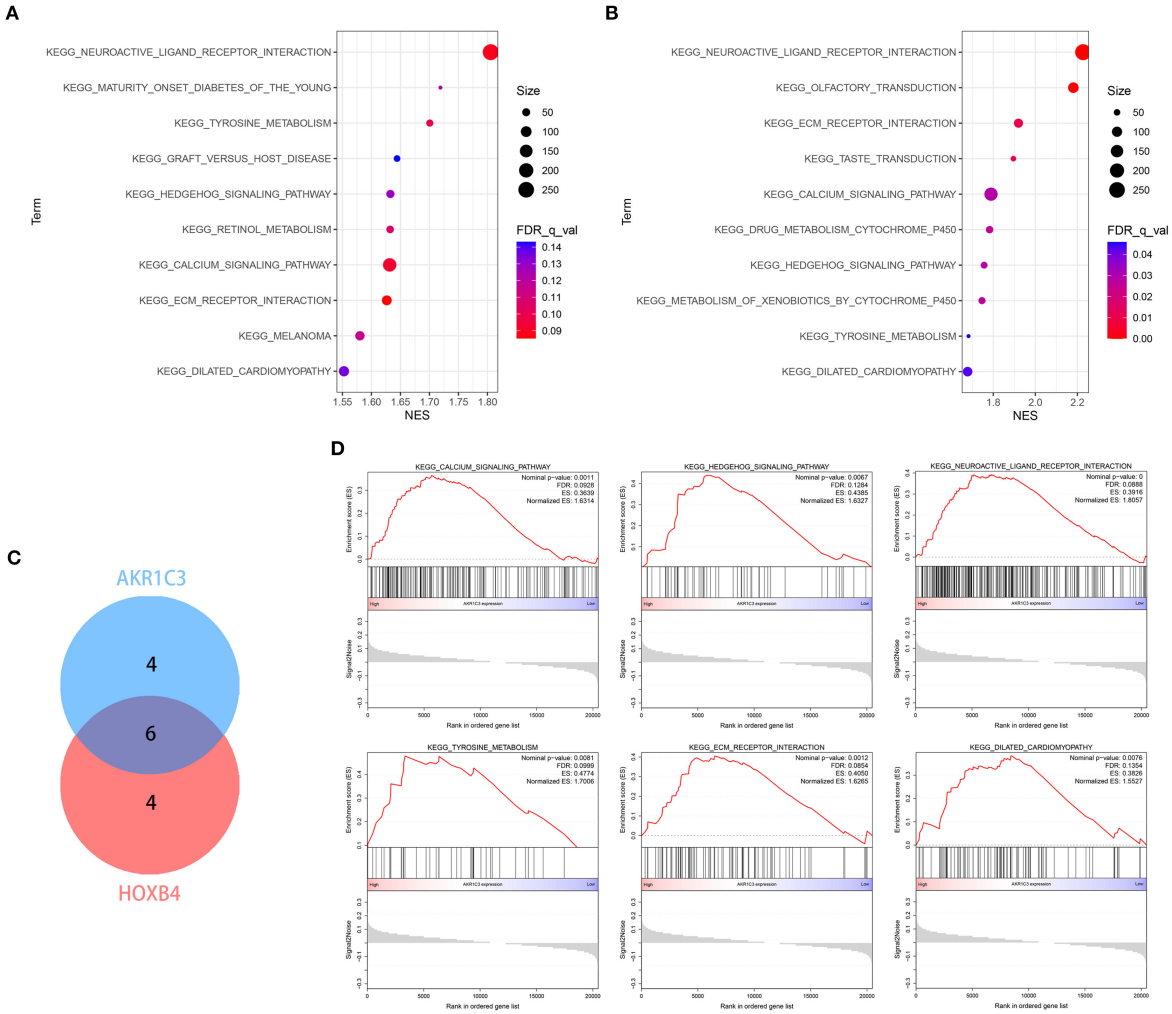
The enrichment analysis *via* GSEA. **(A,B)** The top 10 related pathways for AKR1C3 **(A)** and HOXB4 **(B)**. **(C)** Venn plot indicating six overlapped pathways. **(D)** The overlapped pathways were mostly correlated with cardiovascular diseases. GSEA, gene set enrichment analysis.

Single-gene GSEA strategy was utilized to detect the related biological pathways with NOM *p*-value < 0.05 and FDR *Q*-value < 0.25. The GSEA results are shown in Supplementary Tables 7, 8. The top 10 pathways related to AKR1C3 and HOXB4 are shown in Figures 10A,B, respectively. We found six pathways that were overlapped (Figure 10C), partly indicating the tight association between AKR1C3 and HOXB4. Among these overlapped pathways, most have been reported to be involved in the development of cardiovascular diseases (Figure 10D).

### Identification of Novel Potential Compounds Targeting the HOXB4–AKR1C3 Axis

**Figure 11:**
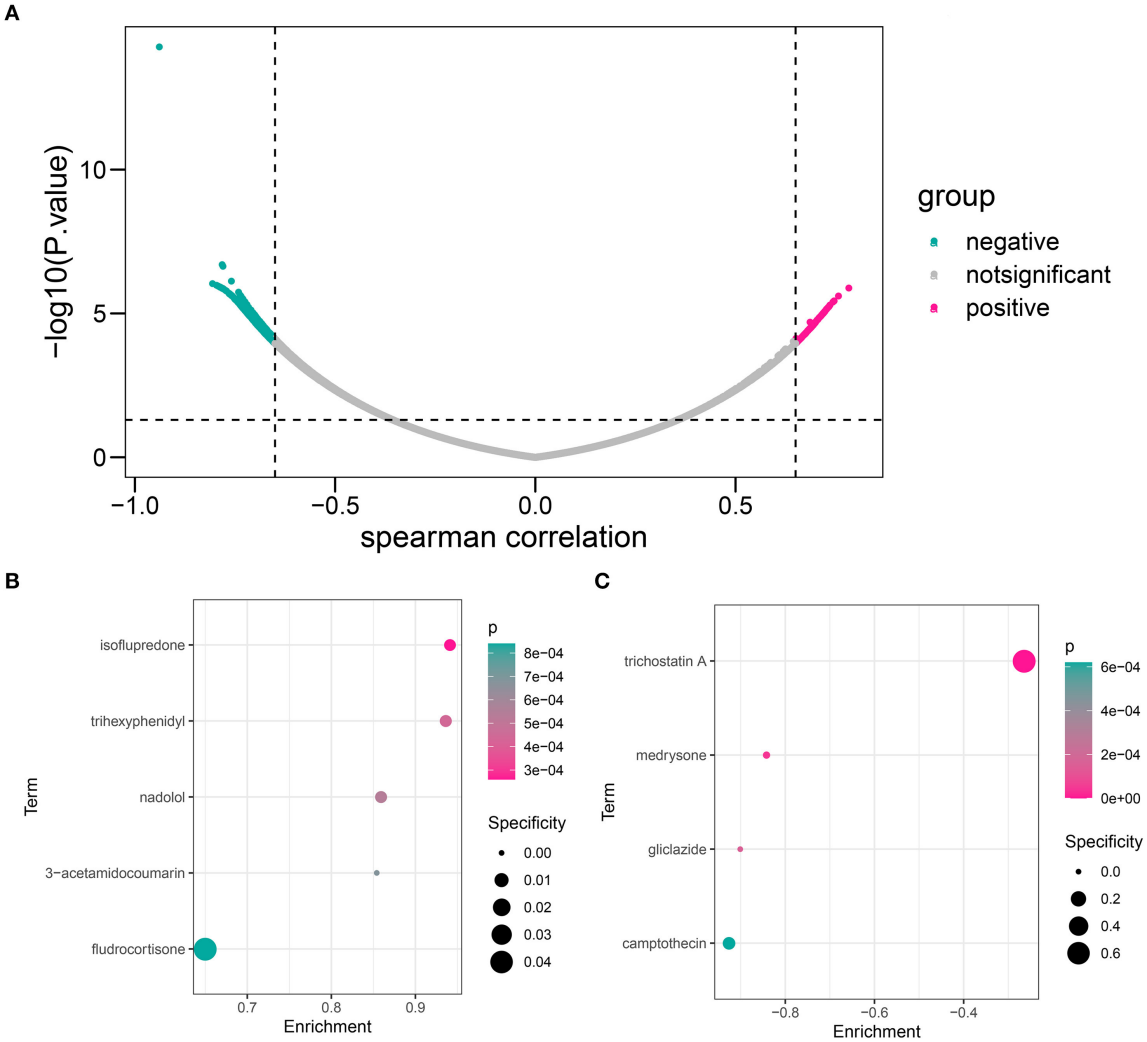
The candidate compound targeting the HOXB4–AKR1C3 axis. **(A)** Volcano map showing that a total of 536 genes, including 136 positively correlated and 400 negatively correlated, were associated with a logical score. **(B)** The compounds which might target the positively correlated genes. **(C)** The compounds which might target the negatively correlated genes.

First, we calculated the Spearman correlation coefficients between each gene and logical score in the GSE48060 dataset. The genes with |*r*| > 0.65 and *p* < 0.05 were chosen as the related genes (Supplementary Table 9; Figure 11A). CMap database (https://portals.broadinstitute.org/cmap/) was used to predict the candidate small molecular drugs targeting the HOXB4–AKR1C3 axis (Supplementary Table 10). Figures 11B,C show the possible compounds targeting the positively correlated genes and negatively correlated genes with *p* < 0.001 filtering, respectively.

## Discussion

Despite advancement in primary prevention and treatment techniques in recent years, AMI remains one of the most common cardiovascular diseases with highest mortality (14, 15). Earlier diagnosis and treatment remain one of the most effective methods to reduce the social burden that AMI brings. Nowadays, dozens of studies focused on the application of gene markers, providing new clues for understanding the molecular mechanism of AMI pathogenesis (16, 17)—for example, Wang et al. firstly identified miR-208a as the potential marker in plasma for AMI (18), and subsequent studies reported that miR-208a enhanced the cardiac functions by regulating the PDE4D/PRKAR1α/PKA phosphoprotein pathway (19) as well as promoting the apoptosis of ischemic cardiac myocytes (20), providing novel treatment targets. It has been reported that ferroptosis played an unneglected role in the pathogenesis of AMI, so screening the potential biomarker correlated with ferroptosis is necessary, which may do help to further elucidate the etiology mechanisms of AMI from the aspect of ferroptosis.

In the present study, we collected the transcriptome data of human blood sample from GEO datasets. To expand the sample size, we systematically merged GSE48060 and GSE97320. A genomic difference analysis revealed that AKR1C3 and NEDD4 were significantly downregulated in the AMI samples. Some researchers have reported the tight relationship between AKR1C3 and AMI (10), but experimental evidence and mechanism exploration are insufficient at the moment, so we chose AKR1C3 as the subject for further study. AKR1C3 was found to be a significant indicator to diagnose AMI through ROC analysis in GSE4806. Furthermore, we discovered that HOXB4, regulating AKR1C3 by acting as the transcription factor, also served as an acceptable diagnosis marker in the training dataset. To better clarify the diagnosis value, we collected 32 blood samples, including 16 AMI samples and 16 control samples, and examined the diagnosis value *via* RT-qPCR, which verified that both genes were ideal diagnostic indicators with high sensitivity and specificity. Subsequently, in order to verify whether HOXB4 could regulate the processes of ferroptosis, a series of ferroptotic markers was detected in the HOXB4-overexpression H9C2 cell lines. To achieve a more accurate diagnostic ability, a nomogram, comprised of AKR1C3 and HOXB4, was successfully established. To validate the efficacy of the nomogram, six different datasets, including GSE34198, GSE42148, GSE48060, GSE60993, GSE61144, and 32 local samples, were collected, and a meta-analysis was conducted. We also detected the correlated functions of the assessment model. WGCNA showed that the genes in tan module, which is highly correlated with the diagnostic model, were mostly enriched in immune-related functions. GSEA indicated that AKR1C3 and HOXB4 were closely correlated with the pathways about myocardial injury. The potential target drugs were also predicted and displayed.

AKR1C3, which belongs to the aldo-keto reductase superfamily acting as NADP(H) oxidoreductases, has been considered as the therapeutic target of multiple malignancies and endocrine diseases (21). A recent study revealed that AKR1C3 was upregulated in erastin-resistant DU-145 prostate cancer cells (22), implying that AKR1C3 may be a suppressor of cell ferroptosis. We also found that the expression of AKR1C3 was downregulated in H9C2 cells treated with RSL3. Previous studies demonstrated that AKR1C3 could affect the contraction and relaxation of vascular smooth muscle *via* regulating the synthesis of prostaglandins (23). The imbalance of prostaglandins was a risk factor for the occurrence of coronary events (24). However, how AKRC1C3 affects the ferroptosis of cardiac myocytes needed to be further detected.

To achieve a deeper understanding of the mechanisms that AKR1C3 enrolled, we predicted the transcription factors and identified HOXB4 as the activator of AKR1C3. Homeobox (HOXB4), a member of homeobox family, has been reported as a transcription factor to PROM1 (25). Previous studies mostly focused on the effect of HOXB4 on hemopoietic cells (26, 27), and a correlation between HOXB4 and AMI has not been reported. Here we used luciferase reporter array to demonstrate the close association between HOXB4 and AKR1C3 and examined its expression value in AMI samples. We found that the overexpression of HOXB4 in cardiomyocytes could suppress ferroptosis. All these results indicated that HOXB4 might be involved in the ferroptotic processes of AMI, but more details were demanded to be clarified.

Several biological signaling pathways were significantly associated with AKR1C3 and HOXB4 *via* GSEA. Among them, some have been reported to be involved in the pathogenesis of AMI—for instance, it was reported that the dysregulation of sonic hedgehog signaling pathway aggravated the cardiac damage of type 1 diabetic mice with MI (28). However, the regulation between HOXB4–AKR1C3 and hedgehog pathway remained unknown. WGCNA helped to identify the risk model-related genes, and some immune-related functions were enriched. It was reported that the hedgehog pathway took part in fat inflammatory, and the chronic low-grade inflammation of adipose tissue has been considered as the key factor of obesity (29). Obesity was usually regarded as the risk factor for AMI. Recent studies revealed the tight correlation between ferroptosis and immunity (6). Therefore, it is possible that the hedgehog pathway could interact with immune cells to induce or inhibit ferroptosis in AMI cases, but more experimental evidence ought to be offered in a future study.

Some drugs were screened to be possibly correlated with the HOXB4–AKR1C3 axis, and some of them have been reported to be widely applied in cardiovascular diseases. Nadolol, which is a beta-adrenergic receptor blocker, has been implemented for the treatment of hypertension, angina, arrhythmia, and other cardiovascular diseases (30). Camptothecin, an anti-tumor drug, often brought cardiovascular toxicity to patients (31). We found that nadolol was enriched in genes positively correlated with diagnostic score (Figure 10B), while camptothecin was negatively associated with diagnostic score (Figure 10C), which helped to disclose the underlying drugs targeting the HOXB4–AKR1C3 axis.

To the best of our knowledge, it is for the first time that ferroptosis-related genes were utilized to discover novel diagnostic molecules of AMI. HOXB4 was firstly reported to have the potential to distinguish AMI from normal cohort and could transcriptionally activate AKR1C3. Though few studies have demonstrated that AKR1C3 might be of high diagnostic value (10), we firstly validated the effectiveness in a local cohort through RT-qPCR. We also constructed and validated a nomogram in multiple datasets, making it easier for clinicians to understand the assessment model, which might be helpful for AMI diagnosis.

However, there are some shortcomings in the present study. On one hand, our peripheral blood samples were all collected from Shunde Hospital, so it is essential to ascertain the diagnostic efficacy in multi-center and large-scale clinical traits. On the other hand, we only detect the related functions through bioinformatical analysis, where more experimental validation was needed.

Generally, we demonstrated that the HOXB4–AKR1C3 axis can act as a potential diagnostic marker of AMI and reliably established a two-gene nomogram, providing novel insights into the underlying mechanisms of AMI.

## Data Availability Statement

The datasets presented in this study can be found in online repositories. The names of the repository/repositories and accession number(s) can be found in the article/Supplementary Material.

## Ethics Statement

The studies involving human participants were reviewed and approved by Ethics Committee of Shunde Hospital of Southern Medical University. The patients/participants provided their written informed consent to participate in this study.

## Author Contributions

YHu and XH conceived and coordinated the study. JL contributed to data analysis, drafted the manuscript, and constructed the qPCR and luciferase reporter assay. YC, MH, WL, and GH contributed to the collection of samples and performed some experiments. TM, ML, and YHua conducted and gave advice on the study. YHu, XH, and YHua reviewed and revised the manuscript. All the authors read and approved the final version of the manuscript.

## Funding

This project was supported by the Guangdong Basic and Applied Basic Research Fund (key project of Guangdong-Foshan Joint Fund; 2019B1515120044), the Science and Technology Innovation Project from Foshan, Guangdong (FS0AA-KJ218-1301-0006), and the Science and Technology Innovation Project from Foshan, Guangdong (FS0AA-KJ218-1301-0010).

## Conflict of Interest

The authors declare that the research was conducted in the absence of any commercial or financial relationships that could be construed as a potential conflict of interest.

## Publisher's Note

All claims expressed in this article are solely those of the authors and do not necessarily represent those of their affiliated organizations, or those of the publisher, the editors and the reviewers. Any product that may be evaluated in this article, or claim that may be made by its manufacturer, is not guaranteed or endorsed by the publisher.

## Supplementary Material

Supplementary Material 1 Examination and approval document from the Ethics Committee of Shunde Hospital of Southern Medical University.

Supplementary Table 1 The differentially expressed genes after combining GSE48060 and GSE97320.

Supplementary Table 2 The predicted transcription factors.

Supplementary Table 3 The correlation between AKR1C3 and candidate transcription factor.

Supplementary Table 4 The results of the luciferase reporter assay.

Supplementary Table 5 The differentially expressed genes in GSE48060.

Supplementary Table 6 The correlation among genes, modules, and logical score.

Supplementary Table 7 The GSEA results for AKR1C3.

Supplementary Table 8 The GSEA results for HOXB4.

Supplementary Table 9 The correlation between each gene and logical score.

Supplementary Table 10 The potential compounds obtained from CMap.

Supplementary Table 11 The primers of the gene for RT-qPCR test.
