# Supplementary material for: AKR1C3 and Its Transcription Factor HOXB4 Are Promising Diagnostic Biomarkers for Acute Myocardial Infarction
Source: Front Cardiovasc Med. 2021 Sep 9;8:694238. doi: 10.3389/fcvm.2021.694238 (PMC8458746; doi:10.3389/fcvm.2021.694238)
Supplement: Supplementary Material 1 — Examination and approval document from the Ethics Committee of Shunde Hospital of Southern Medical University. [file Data_Sheet_1.PDF]

南方医科大学顺德医院医学伦理委员会审查批件

批件号	科研伦审20210207		
项目名称	AKR1C3及其转录因子HOXB4可以作为急性心肌梗死的诊断标志物的研究		
主要研究者	胡允兆	研究类型	基础研究
其他研究者	梁晶晶、黄小惠、曹越、何明立、李伟文、黄国林		
审查类别	初次审查	审查方式	简易审查
审查委员	石向群、杨斌、曾祺		
审查文件	1.南方医科大学顺德医院研究方案<br>2.知情同意书 版本号：1.0 版本日期：2020.11.01		

审查意见：

根据卫生部《涉及人的生物医学研究伦理审查办法》（2016）、WMA《赫尔辛基宣言》和CIOMS《人体生物医学研究国际道德指南》的伦理原则，对该项目的审查均以提交审查文件的为基准进行审查，经本伦理委员会会议审查，该项目符合伦理审查的基本要求，同意按照上述文件开展本项临床研究。

**审查结果**	（√ ）同意<br>（ ）作必要的修正后同意<br>（ ）作必要的修正后重审<br>（ ）不同意<br>（ ）终止或暂停已批准的研究		
年度/定期跟踪审查频率	一年一次	批件有效期	2022/3/1
主任委员签字			
伦理委员会	（盖章）		
批准日期	2021年3月2日		

备注：

1.本临床试验自批件签署日期起1年内实施有效；逾期未实施的，本批件无效。

2.研究应遵循GCP原则和伦理委员会批准的方案开展临床研究，保护受试者的健康与权益。

3.研究过程中若变更主研究者，对临床研究方案、知情同意书材料等任何修改，请申请人提交修正案审查。

4.自同意研究日起（以本批件签署时间为准），按照跟踪审查频率递交年度/定期跟踪审查报告，请在跟踪审查到期1个月递交。

5.发生严重不良事件，请申请人及时提交严重不良事件报告。

6.重大违背或偏离方案应及时提交违背/偏离报告表。

7.申请人暂停或提前终止临床研究，请及时提交暂停/终止研究报告。

8.完成研究，请申请人提交研究完成报告。

声明：本伦理委员会按照中国GCP、ICH GCP和有关法规组成和工作，其审查和工作过程不受任何组织和个人的影响。
